# Supplementary figures and images for: Staphylococcus aureus FtsZ and PBP4 bind to the conformationally dynamic N-terminal domain of GpsB
Source: eLife. 2024 Apr 19;13:e85579. doi: 10.7554/eLife.85579 (PMC11062636; doi:10.7554/eLife.85579)

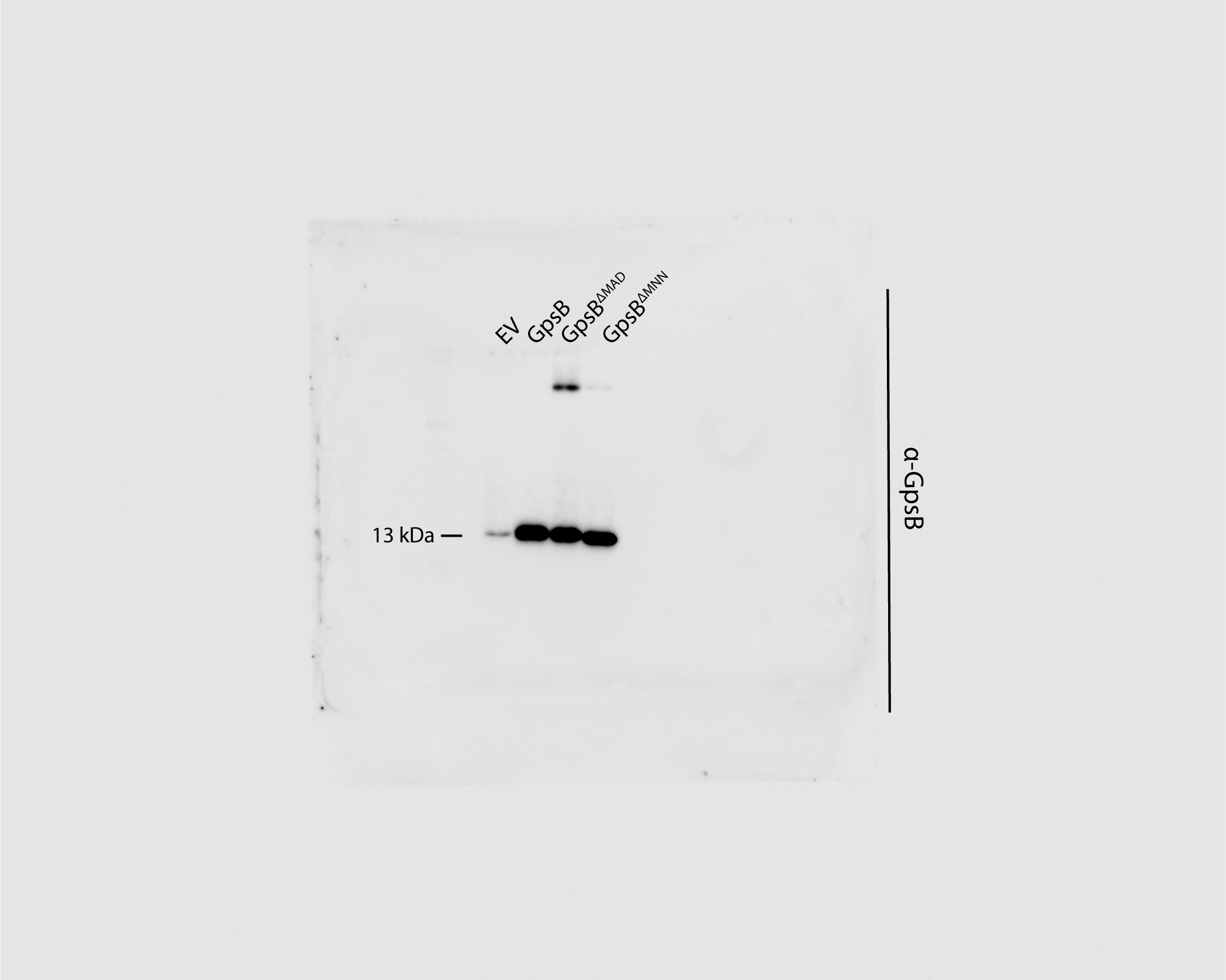

Supplement: Figure 2—figure supplement 1—source data 1. [file elife-85579-fig2-figsupp1-data1.zip › Figure 2-figure supplement 1-source data/anti-GpsB blot.tif]

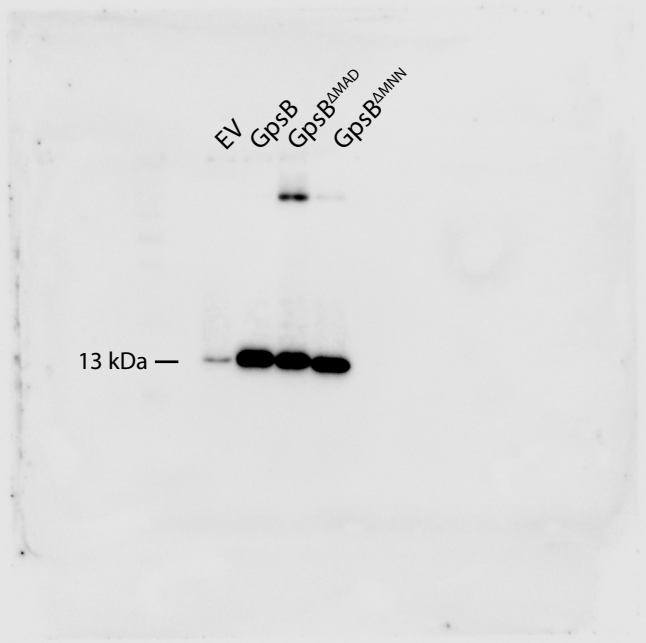

13 kDa —

$\alpha$ -GpsB

Supplement: Figure 2—figure supplement 1—source data 1. [file elife-85579-fig2-figsupp1-data1.zip › Figure 2-figure supplement 1-source data/anti-GpsB blot-labeled.pdf]

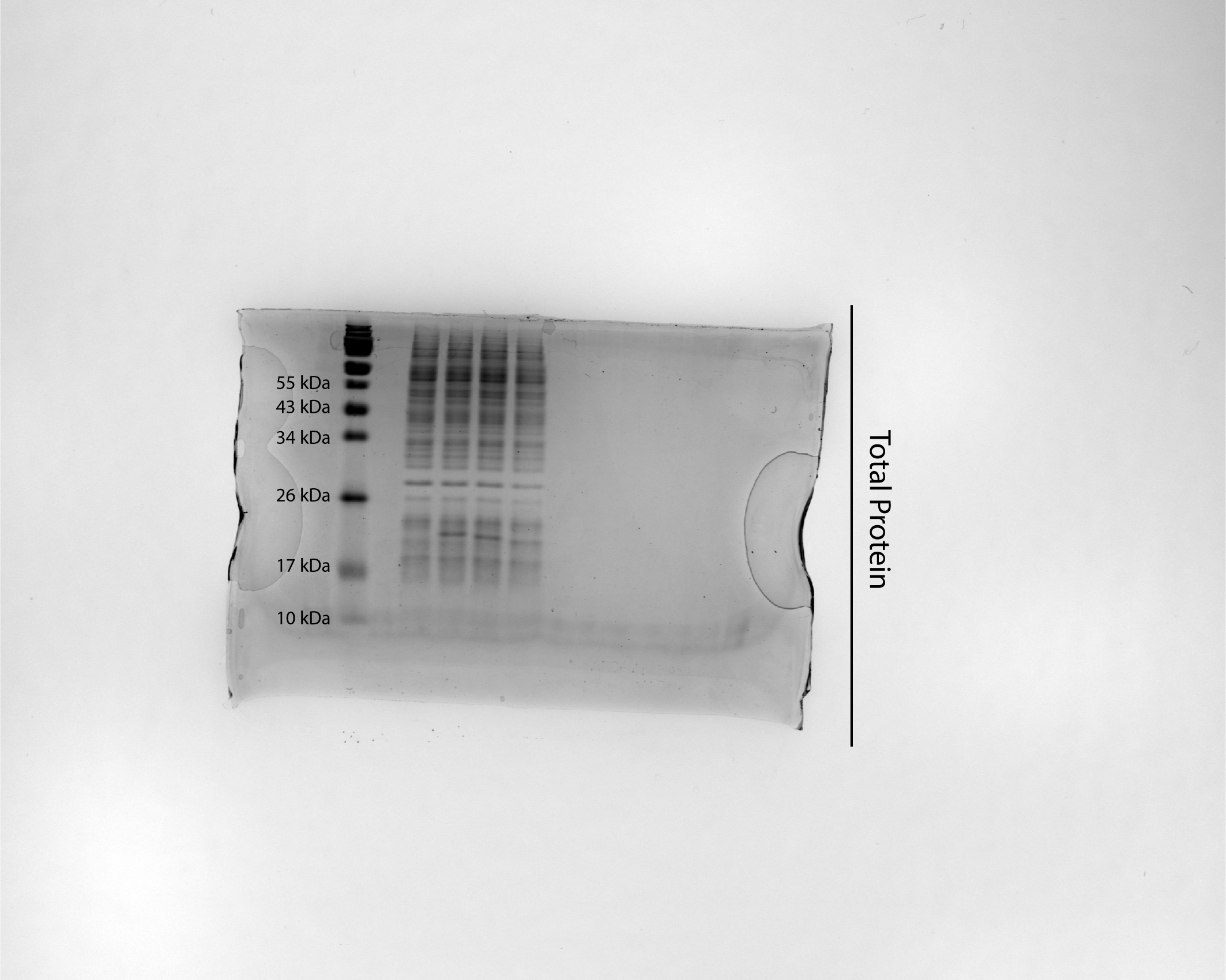

Supplement: Figure 2—figure supplement 1—source data 1. [file elife-85579-fig2-figsupp1-data1.zip › Figure 2-figure supplement 1-source data/Total Protein gel.tif]

Total Protein

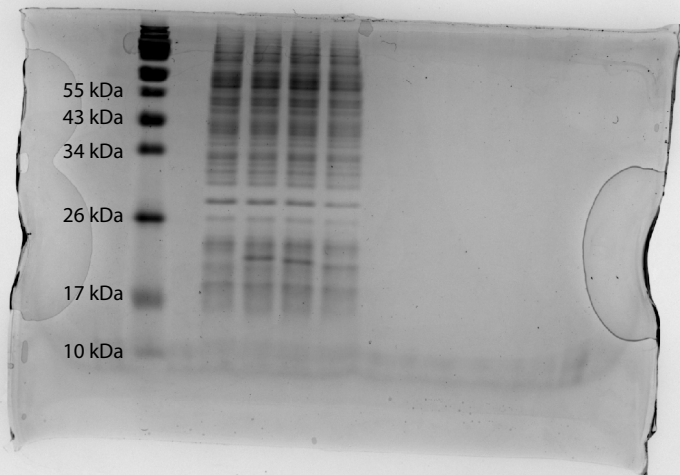

Supplement: Figure 2—figure supplement 1—source data 1. [file elife-85579-fig2-figsupp1-data1.zip › Figure 2-figure supplement 1-source data/Total Protein gel-labeled.pdf]
