# Supplementary material for: Staphylococcus aureus FtsZ and PBP4 bind to the conformationally dynamic N-terminal domain of GpsB
Source: eLife. 2024 Apr 19;13:e85579. doi: 10.7554/eLife.85579 (PMC11062636; doi:10.7554/eLife.85579)
Supplement: Supplementary file 1. — *Values in parentheses indicate those for the highest resolution shell. [file elife-85579-supp1.docx]

| PDB ID | **8E2B** | **8E2C** |
| --- | --- | --- |
| Protein | *Sa* GpsB NTD | *Sa* GpsB NTD + *Sa* PBP4 C-term |
| **Data Collection** |  |  |
| Space Group | P 1 2_1_ 1 | P 1 2_1_ 1 |
| Cell Dimensions |  |  |
| a, b, c (Å) | 53.52, 37.47, 79.25 | 29.35, 73.76, 42.37 |
| α, β, γ (°) | 90, 100.62, 90 | 90, 103.56, 90 |
| Resolution (Å) | 47.88 – 1.95 | 41.19 – 2.40 |
|  | (2.00 – 1.95) | (2.49 – 2.40) |
| R_merge_ | 0.073 (0.190) | 0.095 (0.410) |
| <I>/σ<I> | 9.7 (4.1) | 10.8 (4.9) |
| CC_1/2_ | 0.996 (0.888) | 0.991 (0.885) |
| Completeness (%) | 90.7 (94.8) | 95.7 (96.6) |
| Redundancy | 3.3 (3.2) | 5.2 (5.2) |
| **Refinement** |  |  |
| Resolution (Å) | 40.29 - 1.95 | 36.88 - 2.4 |
|  | (2.02 - 1.95) | (2.49 - 2.40) |
| No. reflections/free | 20762 / 1075 | 6579 / 633 |
| R_work_/R_free_ | 0.188 / 0.240 | 0.227 / 0.248 |
| Clashscore | 4.92 | 5.63 |
| No. Atoms |  |  |
| Overall | 2768 | 1161 |
| Protein | 2335 | 1157 |
| Ligand/Ion | 6 | 0 |
| Water | 427 | 4 |
| B-Factors (Å^2^) |  |  |
| Overall | 24.38 | 40.78 |
| Protein | 22.12 | 40.80 |
| Ligand/Ion | 35.19 | - |
| Solvent | 36.55 | 33.34 |
| RMS Deviations |  |  |
| Bond Lengths (Å) | 0.015 | 0.013 |
| Bond Angles (°) | 1.68 | 1.70 |
| Ramachandran Favored (%) | 99.63 | 99.25 |
| Ramachandran Allowed (%) | 0.37 | 0.75 |
| Ramachandran Outliers (%) | 0.00 | 0.00 |
| Rotameric  Outliers (%) | 3.14 | 2.40 |

Supplementary File 1  **–**  Table of crystallographic statistics

* Values in parentheses indicate those for the highest resolution shell.
