# Supplementary material for: Staphylococcus aureus FtsZ and PBP4 bind to the conformationally dynamic N-terminal domain of GpsB
Source: eLife. 2024 Apr 19;13:e85579. doi: 10.7554/eLife.85579 (PMC11062636; doi:10.7554/eLife.85579)
Supplement: Supplementary file 2. — Concentration response sensorgrams are shown in Figure 4—figure supplement 2. [file elife-85579-supp2.docx]

|  | *Sa* GpsB^WT^_FL_ | *Sa* GpsB^ΔMAD^_FL_ |
| --- | --- | --- |
| *Sa* FtsZ (325-390) | 40.21 ± 1.77 µM | 74.01 ± 4.34 µM |
| *Sa* FtsZ (379-390) | 73.63 ± 9.43 µM | >200 µM |
| *Sa* FtsZ (383-390) | 17.76 ± 1.25 µM | 59.68 ± 4.49 µM |
| *Sa* PBP4 (423-431) | 48.61 ± 1.38 µM | >200 µM |

Supplementary File 2  **–**  SPR dissociation constants (K_D_) of *Sa* FtsZ and *Sa* PBP4 derived peptides for *Sa* GpsB^WT^_FL_ and *Sa* GpsB^ΔMAD^_FL_. Concentration response sensorgrams are shown in Supplementary Figure 7.
