# Supplementary material for: Staphylococcus aureus FtsZ and PBP4 bind to the conformationally dynamic N-terminal domain of GpsB
Source: eLife. 2024 Apr 19;13:e85579. doi: 10.7554/eLife.85579 (PMC11062636; doi:10.7554/eLife.85579)
Supplement: Supplementary file 3. [file elife-85579-supp3.docx]

| **Species** | **Strain** | **Genotype** | **Source** |
| --- | --- | --- | --- |
| *B. subtilis* | PY79 | Wildtype | 1 |
| *B. subtilis* | GG7 | *amyE::P_hyperspank_-gpsB^SA^ spcR* | 2 |
| *B. subtilis* | GG8 | *amyE::P_hyperspank_-gpsB^SA^-gfp spcR* | 2 |
| *B. subtilis* | GG18 | *amyE::P_hyperspank_-gpsB^BS^ spcR* | 2 |
| *B. subtilis* | GG19 | *amyE::P_hyperspank_-gpsB^BS^-gfp spcR* | 2 |
| *B. subtilis* | LH115 | *amyE::P_hyperspank_-gpsB^SA ΔMNN^ spcR* | This study |
| *B. subtilis* | LH116 | *amyE::P_hyperspank_-gpsB^SA ΔMNN^-gfp spcR* | This study |
| *B. subtilis* | LH119 | *amyE::P_hyperspank_-gpsB^SA ΔMAD^ spcR* | This study |
| *B. subtilis* | LH126 | *amyE::P_hyperspank_-gpsB^SA ΔMAD^-gfp spcR* | This study |
| *B. subtilis* | DB44 | *bkdB::Tn917::amyE::P_hyperspank_-gpsB^SA^-gfp ermR; amyE::P_hyperspank_-gpsB^SA^ spcR* | This study (made from LH73; Hammond et al 2022 ^3^ and GG7; ^4^) |
| *B. subtilis* | DB45 | *bkdB::Tn917::amyE::P_hyperspank_-gpsB^SA^-gfp ermR; amyE::P_hyperspank_-gpsB^SA ΔMNN^ spcR* | This study (made from LH73; Hammond et al 2022^3^ and pLH56) |
| *B. subtilis* | DB46 | *bkdB::Tn917::amyE::P_hyperspank_-gpsB^SA^-gfp ermR; amyE::P_hyperspank_-gpsB^SA ΔMAD^ spcR* | This study (made from LH73; Hammond et al 2022^3^ and pLH58) |
| *B. subtilis* | LH78 | *bkdB::Tn917::amyE::P_hyperspank_-gpsB^SA^-gfp ermR; amyE::P_hyperspank_-gpsB^SA ΔLEE^ spcR* | 3 |
| *S. aureus* | PES5 | SH1000 with pCL15 Empty Vector | 2 |
| *S. aureus* | PE355 | RN4220 with pCL15 Empty Vector | 2 |
| *S. aureus* | PES6 | SH1000 pCL15 backbone *P_spac_-gpsB^SA^-gfp* | 2 |
| *S. aureus* | GGS2 | RN4220 pCL15 backbone *P_spac_-gpsB^SA^-gfp* | 2 |
| *S. aureus* | PES13 | SH1000 pCL15 backbone *P_spac_-gpsB^SA^* | 2 |
| *S. aureus* | GGS1 | RN4220 pCL15 backbone *P_spac_-gpsB^SA^* | 2 |
| *S. aureus* | LH132 | SH1000 pCL15 backbone *P_spac_ -gpsB^SA ΔMNN^-gfp* | This study |
| *S. aureus* | LH128 | RN4220 pCL15 backbone *P_spac_-gpsB^SA ΔMNN^ -gfp* | This study |
| *S. aureus* | LH134 | SH1000 pCL15 backbpone *P_spac_-gpsB^SA ΔMNN^* | This study |
| *S. aureus* | LH127 | RN4220 pCL15 backbone *P_spac_-gpsB^SA ΔMNN^* | This study |
| *S. aureus* | LH133 | SH1000 pCL15 backbone *P_spac_-gpsB^SA ΔMAD^-gfp* | This study |
| *S. aureus* | LH130 | RN4220 pCL15 backbone *P_spac_-gpsB^SA ΔMAD^-gfp* | This study |
| *S. aureus* | LH135 | SH1000 pCL15 backbpone *P_spac_-gpsB^SA ΔMAD^* | This study |
| *S. aureus* | LH129 | RN4220 pCL15 backbone *P_spac_-gpsB^SA ΔMAD^* | This study |
| *S. aureus* | LM74 | RN4220 Δ*gpsB* pCL15 Empty Vector | This study |
| *S. aureus* | LM75 | RN4220 Δ*gpsB* pCL15 backbone *P_spac_-gpsB^SA^* | This study |
| *S. aureus* | LM76 | RN4220 Δ*gpsB* pCL15 backbone *P_spac_-gpsB^SA ΔMAD^* | This study |
| *S. aureus* | LM77 | RN4220 Δ*gpsB* pCL15 backbone *P_spac_-gpsB^SA ΔMNN^* | This study |
| *E. coli* | BTH101 | *Adenylate cyclase deficient reporter strain for BACTH; F′, cya-99, araD139, galE15, galK16, rpsL1 (Str^R^), hsdR2, mcrA1, mcrB1, relA1* | 5 |
| *E. coli* | LH40 | pEB355 backbone *gpsB^SA^* | 3 |
| *E. coli* | LH39 | pEB354 backbone *gpsB^SA^* | 3 |
| *E. coli* | LH164 | pEB355 backbone *gpsB^SA ∆MAD^* | This study |
| *E. coli* | MA1 | pEB354 backbone *gpsB^SA ∆MAD^* | This study |
| *E. coli* | LH170 | pEB355 backbone *gpsB^SA ∆MNN^* | This study |
| *E. coli* | LH168 | pEB354 backbone *gpsB^SA ∆MNN^* | This study |
| *E. coli* | PE87 | pUT25-zip | 5 |
| *E. coli* | PE88 | pUT18-zip | 5 |
| *E. coli* | PE84 | pEB355 | 5 |
| *E. coli* | PE83 | pEB354 | 5 |
| *E. coli* | pLH62 | pCL15 backbone *P_spac_-gpsB^SA ΔMAD^* | This study |
| *E. coli* | pLH59 | pCL15 backbone *P_spac_-gpsB^SA ΔMNN^* | This study |
| *E. coli* | pLH63 | pCL15 backbpone *P_spac_-gpsB^SA ΔMAD^-gfp* | This study |
| *E. coli* | pLH60 | pCL15 backbone *P_spac_-gpsB^SA ΔMNN^-gfp* | This study |
| *E. coli* | PE630 | BL21(λDE3)::ΔclpP strain containing pET28a *P_IPTG_-his-ftsZ^SA^* | 2 |
| *E. coli* | EDB01 | BL21-DE3 strain containing pET28a *P_IPTG_-his-ftsZ^SAΔC6^* | This study |
| *E. coli* | PE401 | BL21-DE3 strain containing pET28a *P_IPTG_-gpsB^SA^-his* | 2 |
| *E. coli* | SK7 | BL21-DE3 strain containing pET28a *P_IPTG_-his-ftsZ^SA-CTT^* (C-term 66 aa) | This study |
| *E. coli* | pLH56 | pDR111 backbone *P_spac_-gpsB^SA ΔMNN^* | This study |
| *E. coli* | pLH58 | pDR111 backbone *P_spac_-gpsB^SA ΔMAD^* | This study |
| *E. coli* | pLM30 | pCAS^SA^ derivative for *gpsB* deletion | This study; ^6^ ( |

Supplementary File 3  **–** The genotypes of strains used in the cell-based studies.

References

1. Youngman P, Perkins JB, Losick R. Construction of a cloning site near one end of Tn917 into which foreign DNA may be inserted without affecting transposition in Bacillus subtilis or expression of the transposon-borne erm gene. Plasmid. 1984;12(1):1-9. Epub 1984/07/01. PubMed PMID: 6093169.

2. Eswara PJ, Brzozowski RS, Viola MG, Graham G, Spanoudis C, Trebino C, et al. An essential Staphylococcus aureus cell division protein directly regulates FtsZ dynamics. Elife. 2018;7. Epub 2018/10/03. PubMed PMID: 30277210; PubMed Central PMCID: PMCPMC6168285.

3. Hammond, L. R. *et al.* GpsB coordinates cell division and cell surface decoration by wall teichoic

acids Staphylococcus aureus. *Microbiol Spectrum* **10**, e0141322 (2022).

4. Gamba, P., Veening, J. W., Saunders, N. J., Hamoen, L. W. & Daniel, R. A. Two-step assembly dynamics of the Bacillus subtilis divisome. *J Bacteriol* **191**, 4186-4194 (2009).

5. Battesti A, Bouveret E. The bacterial two-hybrid system based on adenylate cyclase reconstitution in Escherichia coli. Methods. 2012;58(4):325-34. Epub 2012/07/31. PubMed PMID: 22841567.

6. Chen, W., Zhang, Y., Yeo, W. S., Bae, T. & Ji, Q. Rapid and Efficient Genome Editing in

Staphylococcus aureus by Using an Engineered CRISPR/Cas9 System. *J Am Chem Soc* **139**, 3790-3795

(2017).
