# Supplementary material for: Staphylococcus aureus FtsZ and PBP4 bind to the conformationally dynamic N-terminal domain of GpsB
Source: eLife. 2024 Apr 19;13:e85579. doi: 10.7554/eLife.85579 (PMC11062636; doi:10.7554/eLife.85579)
Supplement: Supplementary file 4. [file elife-85579-supp4.docx]

| Primer | Sequence (5’ to 3’) |
| --- | --- |
| oP36 | AAAAAGCTTACATAAGGAGGAACTACTATGTCAGATGTTTCATTGAAATTATCAGCA |
| oP37 | AAAGCTAGCTTTACCAAATACAGCTTTTTCTAAGTTTGA |
| oP38 | AAAGCATGCTTATTTACCAAATACAGCTTTTTCTAAGTTTGA |
| oP46 | AAAGCTAGCATGAGTAAAGGAGAAGAACTTTTC |
| oP24 | GCCGCATGCTTATTTGTATAGTTCATCCATGCC |
| BTH11 | AATAAGAATTCATGTCAGATGTTTCATTGAAATTATCAGC |
| BTH12 | GCTGTATTTGGTAAATAACTCGAGTTATT |
| oLHgblock1 | GCCCCATGTCAGATGTTTCATTGAAATTATCAGCAAAAGATATTTATGAAAAAGATTTTGAAAAAACGATGGCTCGTGGCTATAGAAGAGAAGAAGTAGATGCATTTTTAGATGACATTATTGCTGATTATCAAAAAATGGCCGATGAAGTTGTAAAATTATCAGAAGAGAATCATAAACTTAAAAAAGAATTAGAAGAATTAAGACTACGTGTAGCAACATCAAGACCTCAGGACAATAAAAGTTTTTCTTCGAATAATACAACAACAAATACATCTTCAAATAATGTAGATATTTTAAAACGTATTTCAAACTTAGAAAAAGCTGTATTTGGTAAATAAGCCCC |
| oDB9 | AATAACATATGATGTTAGAATTTGAACAAGGATTTAATCATTTAGCG |
| oDB10 | AATAACTCGAGTTAACGTCTTTCTTCTCTATTTCTAATGAAGCTAGG |
| oP228 | AAACATATGAAATCTGGTAGCACTGGATTCGGAACAAGC |
| oP229 | AAAGGATCCTTAACGTCTTGTTCTTCTTGAACGTCTTTCTTCTC |
| oLM25 | gaaaTTTGAAAAAACGATGGCTCG |
| oLM26 | aaacCGAGCCATCGTTTTTTCAAA |
| oLM27 | TTTGAGATCTGTCCATACCCATGGTCTAGAGAGTGTTAAAACATGACGAATACAGTATATTGTG |
| oLM28 | GTCATTATTAGTAAGACAGTTAAACTTTTGTATTTAGTAATCATTAGAAACTTTGATCTTCAGACCAC |
| oLM29 | GTGGTCTGAAGATCAAAGTTTCTAATGATTACTAAATACAAAAGTTTAACTGTCTTACTAATAATGAC |
| oLM30 | AAGATACAGGTATATTTTTCTGACTCGAGCTGGCACACTATGTAGTGTTGATTTAACGC |
| oLM31 | cctatggaaaaacgccagcaacgcggcctttttacggttcc |
| oLM32 | gtaagtaaaacacttactaattctcatttaatcaatgc |
| gLM1 | TTACTAAATACAAAAGTTTAACTGTCTTACTAATAATGACTATGTTATAATTTTAAAAGTGATATTTTGGGTAATCGCTATATTATATAGAGGAAAGTCCATGCTCACACAGTCTGAGATGATTGTAGTGTTCGTGCTTGATGAAACAATAAATCAAGGCATTAATTTGACGGCAATGAAATATCCTAAGTCTTTCGATATGGATAGAGTAATTTGAAAGTGCCACAGTGACGTAGCTTTTATAGAAATATAAAAGGTGGAACGCGGTAAACCCCTtGAGTGAGCAATCCAAATTTGGTAGGAGCACTTGTTTAACGGAATTCAACGTATAAACGAGACACACTTCGCGAAATGAAGTGGTGTAGACAGATGGTTATCACCTGAGTACCAGTGTGACTAGTGCACGTGATGAGTACGATGGAACAGAACATGGCTTATAGAAATATCACTACTAGTTTAGCTCTCCTAGATGATGGAGAGCTTTTTTCATGAAAAGAACACTTAAAATTAACGCCTTGTCTTGATATAATGACACTGCCTTGTTTTAAAATAGTAAGCGGATGCGTTAATGTATCAGCGATTAAATTTGTTGGAAATGTATAAAAAACACAAGCTAAGAATAAAATACCTGTATAAAAGGAGAATCATATATGTTTCAATTACTTGCAGTTTGTCCGATGGGATTAGAAGCTGTTGTTGCTAGGGAAATTCAAGAATTAGGCTATGAAACAAATGTTGAAAATGGTCGTATATTTTTTGAAGGAGACGCAAGTGCAATTGTAAAGGCAAATTTATGGTTGCGCACAGCAGACCGAATCAAAATTGTTGTTGGACGTTTTAACGCAACAACGTTTGACGAATTATTCGAACAAACCAAAGCGCTCCCTTGGGAATCTATAATTGATAAAGAGGGTAACTTCCCAGTTCAAGGTAGAAGCGTTAAATCAACACTACATAGTGTGCCAG |

Supplementary File 4  **–** The oligonucleotide and geneblock sequences used in the cell-based studies.
